# Research Needs for Implementing Cancer Prevention and Early Detection in Developing Countries: From Scientists' to Implementers' Perspectives

**DOI:** 10.1155/2019/9607803

**Published:** 2019-05-07

**Authors:** Raúl Murillo, Claudia Robles

**Affiliations:** ^1^Centro Javeriano de Oncología – Hospital Universitario San Ignacio – Bogotá, Colombia; ^2^Prevention and Implementation Group – International Agency for Research on Cancer – Lyon, France; ^3^Unit of Infections and Cancer (UNIC), Cancer Epidemiology Research Programme - Institut Catala d' Oncologia – Barcelona, Spain

## Abstract

Implementation of evidence-based cancer prevention and early detection in low- and middle-income countries (LMIC) is challenging. Limited and inappropriate introduction of novel alternatives results in an equity gap whereby low-income populations receive a lower benefit. Implementation research represents an opportunity to foster the adoption and expansion of evidence-based cancer control strategies; however, scientific development in high-income countries does not necessarily fulfill the particular needs of LMIC in the field. A review on the link between implementation research and practice, the tension between theory and pragmatism, the conflict around implementation research methods, and determinants of research priority definition was carried out by considering the perspective of cancer prevention and early detection implementers in LMIC. Basic principles and alternatives to overcome implementation research challenges in these settings are discussed.

## 1. Introduction

**Figure 1:**
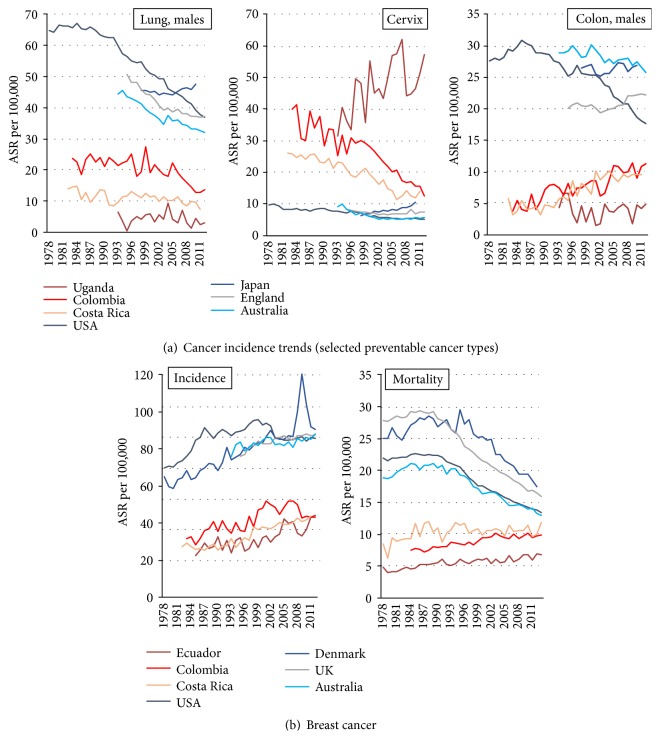
*Incidence and mortality trends for preventable cancers in selected developed and developing countries*. (a) Incidence trends for cancers with evidence-based preventive strategies (risk-factor control or treatment of precancerous lesions). (b) Incidence and mortality trends for breast cancer as a function of evidence-based screening. Mortality data correspond to national registration. Incidence data as follows: Australia, England, Denmark, and Costa Rica correspond to national data; Colombia, Cali; Ecuador, Quito; Japan, 4 registries; Uganda, Kampala; USA, SEER 9 registries. Sources: (1) WHO-IARC, Cancer Mortality Database (last update December 2014), and (2) IARC, Cancer Incidence in Five Continents (CI5*plus*).

Cancer is a leading cause of morbidity and mortality worldwide [1, 2]. Although several countries have achieved significant reductions in the cancer burden [3], progress in cancer control is largely unequal. The incidence of preventable malignancies such as lung, cervical, and colorectal cancer has decreased in high-income countries while remaining unchanged or increasing in low- and middle-income countries (LMIC). Similarly, breast cancer screening has lowered mortality rates only in high-income nations, whereas mortality from breast cancer continues to increase in most developing countries (Figure 1).

Several factors influence the successful implementation of novel cancer control alternatives, including health system organization as well as social and economic determinants. Accordingly, the ability and capacity to properly introduce new technologies and programmatic approaches in dissimilar contexts have become crucial in reducing disease burden disparities and in cancer control in general terms. During the last decade, implementation research has been proposed as a means to foster the adoption and expansion of evidence-based cancer control strategies; thus, it is currently a field of intensive activity with growing support by funding and governmental institutions [4].

Several theoretical frameworks have been developed in the field aimed at providing structured guidance to practice; however, implementation research and practice are still facing relevant challenges such as the differential nature of health problems and control interventions and the variability in requirements for specific settings. Indeed, cancer biology (by tumor type), the natural history of the disease (preinvasive/invasive), and the diversity of interventions for cancer prevention and early detection significantly impact program organization and the economy of health systems, thus increasing the complexity of the implementation process and associated research.

Theory and methods of implementation research have been extensively discussed and summarized [5, 6], but most of the literatures in the field come from developed countries and academic centers, with limited participation of implementers from low- and middle-income countries (LMIC) [7]. Although the perception may be subtle, developing science around the implementation process is different from using science to help implement public health interventions. Accordingly, the purpose of this paper is to discuss the research needed to invigorate implementation of population-based cancer prevention and early detection interventions in LMIC.

## 2. The Link between Implementation Practice and Research

**Figure 2:**
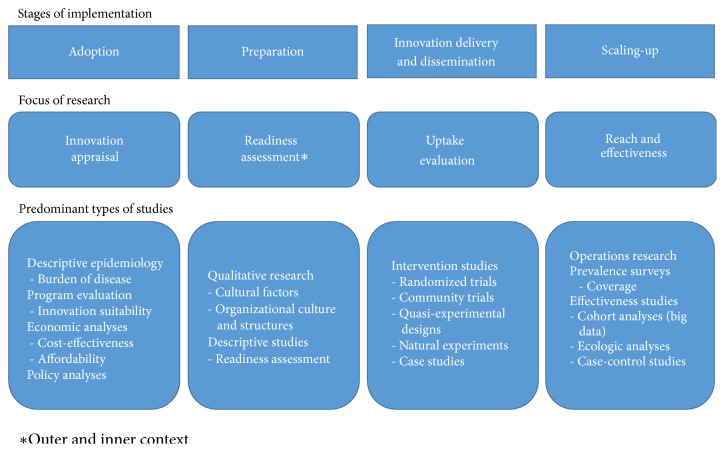
*Research needs for implementation of cancer prevention and early detection interventions. *Adoption refers to the decision-making process. As a subject of research readiness, it precedes innovation delivery; otherwise, it could be integrated. *∗*Outer and inner contexts as defined by Aarons GA et al. [7].

Most theoretical frameworks recognize the need of several steps for integrating evidence-based practices in a given scenario (Table 1); however, implementation science is frequently restricted to the study of what happens after the decision to adopt a new alternative and before its sustained use (Stage/Phase 4 in Table 1) [5]. Undoubtedly, implementation without adoption is not possible, and, similarly, implementation without subsequent scaling-up will not lead to the desired population effect; thus, from a population-based perspective, a comprehensive approach to conducting research on the whole process by considering adoption, dissemination, delivery of interventions, and scaling-up as phases on the same path is key (Figure 2). Given the focus on the delivery phase (assumed as the implementation phase in most frameworks), the study of differences between the abovementioned stages is scarce, where special challenges for each one, as well as differential actors and contextual factors, may play a relevant role in the successful integration of research results into public health practice [8].

The decision (adoption or adaptation) to introduce new technologies or programs in the public health setting is shaped by several organizational and external factors where the availability of strong scientific evidence may be neither compulsory nor sufficient. From the perspective of the innovation decision process, attaining a proper level of knowledge (from scientific evidence) is the first step toward adoption, but the final decision is influenced by other considerations such as envisioned relative advantages, compatibility with current structures and processes, level of complexity, testability (trialability), and observability [15]. Yet, several decisions on public policies are not evidence-based but are guided by the personal circumstances and characteristics of decision-makers [7]. There is scant evidence on the topic, but data from small-scale initiatives indicate that better-informed decisions based on research results are more likely to lead to successful implementation [16].

Hence, decision-making in the public sector is complex, and, in many cases, research to help the adoption decision must be the initial step to foster implementation (types of research at this stage are suggested in Figure 2). In addition, final adoption could also be preceded and further informed by some degree of experimentation with the targeted innovation in the implementation research context, although this is not common practice currently.

Preparedness for implementation of public health interventions is extremely relevant [11, 12], but analyses of readiness in the current literature are mainly circumscribed to management of emergencies, disasters, and emerging diseases. Furthermore, most implementation frameworks consider preparedness as part of delivery; however, the introduction of new technologies and changes in public health programs might require major preparatory interventions in advance, such as modification of legal or regulatory frameworks, definition of funding mechanisms, and review of organizational structures [8, 11]. Indeed, planning activities for delivery such as training, education, and communication require proper prior assessment [11, 12], and research tools could be necessary for that purpose if no accurate information is available, a common situation when no strong health and information systems are present. A recent review identified 30 tools for readiness assessment, all of them addressing capacity in five domains [17].

In summary, the implementation of population-based interventions should consider at least four different stages, namely, decision-making or adoption, preparation, delivery of innovations, and scaling-up [8] (Figure 2). Rabin BA et al. suggest that implementation research seeks to understand processes and factors related to the successful incorporation of evidence-based practices in a given scenario [18]; in this sense, research for implementing cancer prevention and early detection should involve a broad range of topics from the adoption process to expansion of the intervention. This approach does not correspond to a strict definition of implementation science, but it is better aligned with the role of research in understanding the context and supporting integration and scale-up of innovations within health systems at the national level [19]. Not every step or action in the implementation process should or could be a subject of research, but a comprehensive understanding of the process would help to better identify the research needed to foster the introduction of novel cancer control alternatives.

## 3. Theory and Pragmatism for Implementation Research in Developing Countries

The development of implementation science enhances the interpretation of research findings and promotes the inclusion of essential components of the implementation process when conducting studies [6]. Accordingly, there are an increasing number of models and frameworks aimed at providing guidance for both implementation practice and research, thus enriching different perspectives and possible approaches in this area. In general, these models can be classified as process models, determinant frameworks, implementation theory, and evaluative frameworks [20].

An analysis of 61 frameworks (including the most frequently used theories in implementation research for health) found the majority to be oriented toward dissemination rather than implementation and focused on interventions at the institutional and community levels rather than focusing on public policy implementation [6]. Despite its high quality, concentration of implementation research at the institutional level (health center, healthcare organization) might become worthless for cancer control if no major progress in adoption of public health policies and scale-up of interventions is observed.

Indeed, the scaling-up of innovative interventions and integration into health systems is a complex task. Introducing changes to health systems involves decision-making on public policy, and, beyond technical capacity, these decisions are influenced by political values and perspectives, particularly in developing countries where political instability is more common [26]. This critical situation is acknowledged by some frameworks when considering policy development and the effect of political contexts on the implementation process [9, 27–30]; accordingly, instead of staged approaches, these frameworks use systemic approaches with complex, multilevel relationships between components.

The strong link between academia, science, and public health practice in developed countries has benefited extensively from theory development, and, consequently, implementation research has played a relevant role in innovation adoption [31]. On the contrary, the difficult socioeconomic conditions, political uncertainty, and lower technical capacity in low resource settings make theoretical frameworks more suitable for evaluative and analytical purposes rather than for designing implementation plans and research. Therefore, the introduction of new technologies or programs in such settings requires eclectic and innovative approaches, and scientific accuracy and theoretical backgrounds should be understood in this context when preparing and judging implementation research proposals.

Despite the challenges facing implementation practice in LMIC, the application of basic principles could help ensure the achievement of wide population effectiveness when conducting research to support the process [32, 33]. Essential elements should include the involvement of implementers, integration with policy and program decision-making, comprehensive understanding of settings and systems, the use of various populations and contexts, and flexibility of methodological approaches to properly answer research questions in real settings [19, 33].

Furthermore, the long induction time of malignant tumors makes sustainability a critical component of preventive interventions [34]. Active involvement of implementers and their integration within the structure of health systems promote sustainable interventions; however, financial, political, social, cultural, and organizational factors make a priori appraisals difficult [35]. A review found that most sustainability studies rely on subjective measures, are retrospective, focus on fidelity and adoption, and do not examine adaptations to context over time [36]. Different frameworks have been proposed to overcome these limitations, including tailored analyses for different types of interventions from an organizational perspective as well as dynamic approaches for analyzing ongoing changes when moving from the research setting to the practice setting (inner or organizational context) and the ecological system (outer or social context) [8, 37–39]. Parra-Cardona R et al. also highlight the need of a shared leadership with local communities and the complex nature of preventive cultures in LMICs [40].

Clearly, not all implementation strategies work for all settings and populations [33, 41], and, consequently, the applicability of implementation research results from restricted scenarios is limited. Hence, conducting implementation research by using different populations and settings will contribute not only to a better assessment of sustainability but also to a better understanding of the determinants of successful implementation and population effectiveness. Moreover, although desirable, a systematic introduction of research components in the implementation process is not feasible for most LMIC; thus, combining strategies, populations, and settings might help enhance the usability of research results and improve implementation of cancer prevention and early detection in settings with a low capacity to perform research.

## 4. Conflicting Methods for Implementation Research

**Figure 3:**
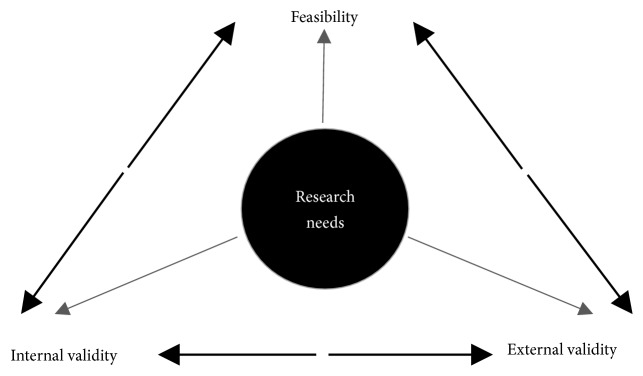
*Driving forces for implementation research in population-based prevention and early detection for cancer control in developing countries*. Research needs denote research questions properly aligned with implementation requirements to advance cancer prevention and early detection.

Study designs and methods of implementation research have been extensively debated, mainly regarding the essential tension between internal and external validity (Figure 3) [42]. Randomized designs are considered the gold standard in health research, as they provide the highest internal validity; however, they require controlled conditions with low to no influence of contextual factors affecting the comparability between groups. Since one of the main objectives of implementation research is to analyze the effect and relevance of contextual factors in the implementation process (external validity) [43, 44], numerous authors doubt the adequacy of randomized trials for evaluating healthcare interventions in real settings, especially for interventions aimed at improving population health in underserved communities [42, 43].

Cluster randomized interventions (allocation of groups) have been used to surmount this limitation; however, these designs require larger sample sizes and constrain the use of mass media campaigns for educational or promotional purposes due to the risk of contamination in control groups. Consequently, individual randomized trials seem to be more appropriate to evaluate implementation strategies on a small scale within a given organization where contextual factors equally affect all groups under evaluation and are not a central component of the study. Anyway, using a control comparable with the subjects of the intervention has been questioned from ethical and technical perspectives in implementation research, since this is the last step in the research process for interventions with already proven efficacy. Alternatively, several options for comparison have been proposed, including step-wedge trials (delayed intervention for the control group), time-series analysis, and simulation by modelling [44, 45].

In addition, a broad range of proposals for integrating research into routine practice has been developed, including traditional approaches such as quasi-experimental designs, community trials, natural experiments, case studies, and new developments like implementation effectiveness research, pragmatic trials, hybrid designs, participatory action research, and so forth [4, 19, 43]. Most of these methods revolve around the delivery and dissemination of innovations within the implementation process; however, a wider approach for research throughout the implementation process requires a broader range of designs and methods, as previously described (Figure 2).

As noted, internal validity, external validity, and feasibility represent major driving forces for implementation research in low-resource settings (Figure 3); the adequate balance between them demands a higher focus on implementation needs and a greater relevance of associated research questions, whereas scientific soundness should be obtained by innovative approaches from single descriptive studies to the development of complex designs. In all cases, implementation research must enhance and accelerate the implementation process and by no means hamper it.

An additional issue is the need for a larger spectrum of reliable outcome variables for a proper assessment of sustainability and scalability of cancer prevention. Research translation from clinical trials to public health programs usually leads to a reduction of intervention efficacy [37, 46]; indeed, a major difference between the two scenarios can be anticipated for interventions with small-magnitude effects in randomized trials or challenging quality assurance requirements. For example, smoking cessation counselling (short and intensive) has proven to induce a statistically significant reduction of tobacco smoking, but the magnitude of the clinical effect in randomized trials ranges between 2% and 16% [47]. Difficulties for sustaining standardization when implemented on a large scale as well as different patient-related factors might induce substantial reductions in effectiveness, leading to results close to the null when implementing smoking cessation programs within health systems. Similarly, cancer screening is a complex intervention in which uptake and fidelity to standard protocols do not ensure high effectiveness given the additional influences of healthcare accessibility, diagnostic test providers, and variability in patients' biological and social backgrounds, among other factors [48]. Therefore, in the case of cancer prevention, the usual implementation outcomes, although necessary, might be insufficient for decision-making or scaling-up.

Using short-term outcomes of cancer prevention and early detection, such as disease stage at diagnosis, detection rates, or biological response, adds value to implementation research by integrating quality and other influential factors that are difficult to measure by other means (Table 2). Although effectiveness research and implementation research are different, given their links, they may benefit if connected [4]. As suggested by some authors, using implementation, service, and health outcomes provides a better framework for understanding the difference between implementation and intervention failures when evaluating population effectiveness [49]. However, using short-term outcomes for cancer prevention and early detection might demand large sample sizes, given their relatively low prevalence (Table 2), thus increasing the challenges of study design in developing countries.

## 5. Implementation Research Needs and Priority Definition

Harmonization between implementation research and practice may be in tension within the comprehensive approach envisioned. While research could help accelerate implementation, an excessive demand for it may possibly become a barrier for introducing new alternatives to cancer control, particularly in low-resource settings; therefore, identifying research needs and defining research priorities through the implementation process are crucial.

**Figure 4:**
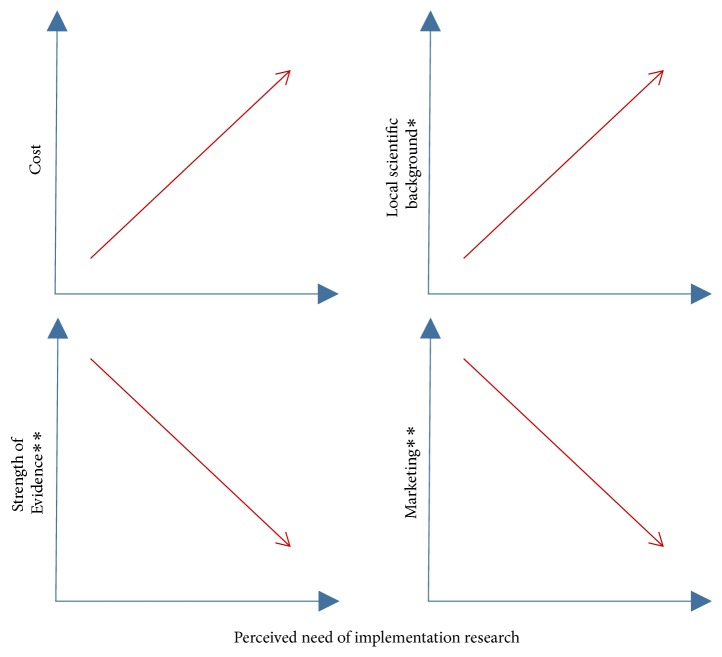
*Possible factors influencing priority definition for implementation research in developing countries. ∗*Scientific capacity is usually linked to greater availability of baseline information for adoption and preparation. Simultaneously, gathering data for these purposes is proposed as the initial opportunity for research in the implementation process when none are available (see Figure 2). *∗∗*Exposure of independent physicians' peers and networks as a result of strong evidence contributes to dissemination in addition to innovation marketing by manufacturers.

Given their proximity to policy decision-making, the perceived needs of implementation research are highly subjective. In general terms, a higher availability of baseline data on the burden of disease and program performance favors identification of knowledge gaps and research needs, and it is usually associated with a higher scientific capacity in the implementation setting (Figure 4). International efforts have considerably improved existing information on cancer occurrence and survival worldwide as part of the basis for decisions on adoption [3, 50, 51], thus reducing research needs in the initial exploratory stage of the implementation process (Figure 2) and shifting priorities to other topics within the same or subsequent phases.

Besides scientific capacity and the availability of baseline data, other factors that could also influence the perceived needs of implementation research are the cost of innovations, strength of associated evidence, and level of innovation marketing (Figure 4). In cancer treatment, the time lag between discoveries and regulatory approvals for new drugs and technologies is shortening rapidly [52, 53], mainly due to increased knowledge on cancer biology and the role of the biotechnology industry in disseminating innovations, stimulated by the growing oncology market. Hence, an independent dynamic has been created, wherein scientific peers and networks play a relevant role in dissemination of innovations, thus shaping professional practices [54, 55]. Population-based interventions for cancer prevention and early detection are less affected by such drivers due to the greater cost for the health system and their dependency on political decisions rather than on individual practices, among other factors. Thus, while marketing hastens innovation adoption, the potential financial impact has the contrary effect and, consequently, perceived needs for implementation research increase.

**Figure 5:**
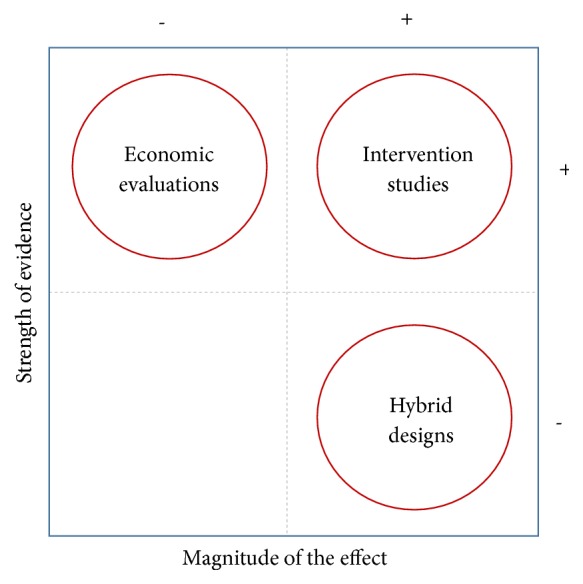
*Illustration of special research approaches to advance implementation according to the strength of scientific evidence. *Signs + and – refer to a higher or lower strength of evidence and magnitude of the effect. The place of a given research approach indicates its suitability in the specific situation resulting from combining the strength of evidence and potential magnitude of the effect. Suitability of studies for a given situation does not restrict their use in other contexts and conditions, and it does not exclude the possibility of combining research approaches in a single study. Interventions studies as described in Figure 2.

Innovations supported by strong evidence may also create an independent dynamic in dissemination of innovations within the scientific community and, consequently, may reduce the perceived needs of implementation research as in the case of marketing (Figure 4); nevertheless, low levels of evidence could motivate interest in special research approaches to prompt implementation of new opportunities for cancer control. As previously described, strong evidence is not compulsory for introducing innovations; however, nowadays, different tools and research approaches are used to face the uncertainty derived from the low strength of scientific evidence when aimed at translation to the public health domain (Figure 5). Impact modelling and economic evaluations could help decision-making by estimating the benefits (and costs) of implementation at the population level, particularly for interventions with reported clinical effects of low magnitude. Similarly, hybrid designs open the possibility of speeding up implementation by conducting effectiveness evaluations while gathering information about the implementation process (or vice versa) [56]. The inclusion of short-term outcomes for cancer control is close to this idea (Table 2), and another proper field for these designs is the study of preventive interventions with low strength of evidence but a large population impact, such as healthy policies for behavioural risk factor control.

For instance, individual weight management programs and surgical and pharmacological treatments for obesity control rely on strong evidence from randomized trials [57]; however, the impact of these interventions has not been translated to the population level. Furthermore, their implementation on a large scale is highly challenging and resource-intensive and, thus, unlikely in developing countries. Despite weaker evidence from clinical studies, interventions for which large-scale implementation is less challenging, such as portion control, reformulation of meals in the food market and school restaurants, or reduced availability of high-calorie food and beverages, might have a greater impact on the population [57]. Both higher- and lower-evidence interventions could benefit from research to compare the effectiveness of obesity control policies by implementing them in different settings while gathering information on the implementation process.

Priority definition for implementation research could also be influenced by funding sources. Funding agencies establish internal objectives within their own theoretical frameworks that may or may not be aligned with implementation research priorities in LMIC; in consequence, the search for funding sources deserves careful attention in order to avoid deviation from primary needs. Furthermore, a large magnitude of external funding for implementation research might conflict with integration of innovations within health systems, because the implementation process should comprise financial sustainability and political commitment from the real setting.

## 6. Conclusions

The development of implementation research and science represents a remarkable opportunity to reduce disparities in cancer control; however, particular conditions of underserved populations and scarcity of resources in most LMIC require a broad perspective within the whole implementation process not restricted to the delivery of innovations or programs.

Informed and evidence-based decisions in public health are highly desirable; however, after the proof of concept, an excessive demand for intensive or complex implementation research could unnecessarily delay or hinder the introduction of new technological and programmatic alternatives given the frequent political uncertainty and lower technical skills in developing countries. Indeed, the diverse character of preventive interventions and the low frequency of short-term cancer-related outcomes increase the complexity of implementation and effectiveness research on cancer prevention and early detection strategies. Sustainability, coverage, and special outcomes required for population-based interventions in cancer control impose additional challenges to underserved communities.

In this context, the role of funding agencies and international organizations becomes essential to harmonize the quality of research and implementation needs. Additionally, some basic principles should be considered to promote better integration between implementation research and practice in lower-resource settings, where flexibility of research methods is particularly relevant to properly responding to specific needs and research questions.

## Figures and Tables

**Table 1:** Phases of the implementation process as described in selected theoretical frameworks.

Models	Proposed stages/phases in the implementation process				
	Stage/Phase 1	Stage/Phase 2	Stage/Phase 3	Stage/Phase 4	Stage/Phase 5
Dissemination and implementation research in health [9]		Dissemination	Adoption	Implementation	Sustainability
RE-AIM [10]	Reach	Effectiveness	Adoption	Implementation	Maintenance
Quality implementation framework [11]		Initial considerations	Creating structure	Ongoing implementation	Improving
Implementation in public service (EPIS) [8]		Exploration	Adoption Decision Preparation	Active implementation	Sustainment
Precede-proceed [12]			Assessment*∗*	Implementation	Evaluation*∗∗*
PARiHS [13]	Evidence	Context	Facilitation	Successful implementation	
Implementation for cancer prevention and control [14]	Controlled and observational studies	Evidence that a technology works	Planning programs	Guiding implementation	Conducting evaluation

The implementation stage is aligned in all models for illustrative purposes. All models in the table correspond to stage models (developed for the health sector), which are different from system models. *∗* represents six phases of assessment: social, epidemiological, behavioral and environmental, educational and organizational, administration, and policy. *∗∗* represents three phases of evaluation: processes, impact, and outcomes.

**Table 2:** Selected population-based implementation studies for cancer prevention and screening.

Study	Field	Intervention	Design	Sample size	Outcomes
Khuhaprema T et al. [21]	Colorectal cancer screening	Fecal occult blood test	Pilot noncontrolled trial	127.301	Participation rate Screening positivity Detection rates

Arrossi S et al. [22]	Cervical cancer screening	Self-collected versus clinician-collected HPV tests	Cluster randomized trial	6,013 in both branches	Screening uptake Screening positivity Detection rates

Murillo R et al. [23]	Breast cancer screening	CBE and mammography versus regular care	Cluster randomized trial	15,838 in both branches	Stage of disease Conservative surgery

Slater JS et al. [24]	Smoking cessation	Direct mail invitation versus opportunistic referral	Nonrandomized trial	5,420 in both groups	Participation rates 7-month smoking abstinence

Moodley I et al. [25]	HPV vaccination	Education and training of stakeholders	Pilot noncontrolled trial	963	Vaccine uptake Side effects

CBE: clinical breast examination.
